# Incidence of clotted haemopericardium in traumatic cardiac arrest in 152 thoracotomy patients

**DOI:** 10.1186/1757-7241-22-S1-P20

**Published:** 2014-07-07

**Authors:** Enikö Manz, Lyndon Nofz, Ain N Norman, Gareth E Davies

**Affiliations:** 1 London’s Air Ambulance, London, UK

## Background

The incidence of haemopericardium in traumatic cardiac arrest is unknown. Treatment for cardiac tamponade has moved from needle pericardiocentesis to open thoracotomy. This small study aims to validate this swing in practice. London’s Air Ambulance (LAA) has been performing emergency thoracotomies for traumatic cardiac arrest since 1993 [1].

## Method

A five year retrospective trauma database search (01/01/08 to 18/09/13) was carried out for all performed clamshell thoracotomies (n=166) by LAA. Presence of clotting, patient demographics, clinical notes and follow-up data was collected and analysed.

## Results

After exclusion of 14 patients 152 patients met inclusion criteria. Of these cases, 61 (40.1%) had no pericardial tamponade, 79 (52.0%) had tamponade and 12 (7.9%) had no available data. Of the patients with confirmed pericardial tamponade, 39 (47.6%) had clotted blood within the pericardial sac. 12 patients (14.6%) it was positively confirmed as fluid blood only. 28 patients (34.1%) there was no description.

## Discussion

Current ATLS and ERC guidelines emphasise the use of emergency thoracotomy in lieu of pericardiocentesis in this population [3]. Our retrospective data shows that a significant percentage of tamponades (47%) are clotted haemopericardium and the shift in trauma management is appropriate.
